# A new protocol (THIS and FAT) for the treatment of filler-induced vascular occlusion: a case series

**DOI:** 10.3389/fmed.2025.1585983

**Published:** 2025-07-30

**Authors:** Shahriar Nazari, Nabil Fakih-Gomez, Nima Hadadian, Foroohe Bayat, Behnam Bohlouli, Cristina Muñoz-Gonzalez, Mohammad Reza Pourani

**Affiliations:** ^1^Department of ENT and Head and Neck Surgery, BMI Hospital, Tehran, Iran; ^2^Department of Facial Plastic and Cranio-Maxillo-Facial Surgery, Fakih Hospital, Khaizaran, Lebanon; ^3^Department of Surgery, Universidad de Salamanca, Salamanca, Spain; ^4^School of Medicine, Tabriz University of Medical Sciences, Tabriz, Iran; ^5^School of Medicine, Ahvaz Jondishapur University of Medical Sciences, Ahvaz, Iran; ^6^Oral and Maxillofacial Surgery, University of Toronto, Toronto, ON, Canada; ^7^Skin Research Center, Shohada-e Tajrish Hospital, School of Medicine, Shahid Beheshti University of Medical Sciences, Tehran, Iran

**Keywords:** hyaluronic acid, ischemia, hyaluronidase, necrosis, fat membrane, nanofat membrane

## Abstract

**Introduction:**

Hyaluronic acid (HA) fillers are generally safe; however, the most significant complication is vascular occlusion. Several therapeutic protocols have been proposed for managing ischemia. De Lorenzi introduced the HDPH protocol, which uses a minimum of 500 IU of hyaluronidase (HYAL) per ischemic area.

**Materials and methods:**

This case series study evaluates the efficacy of a novel protocol, “THIS and FAT,” for managing ischemia resulting from filler-induced vascular occlusion (FIVO). The protocol builds on elements from previous approaches while introducing combination therapies specifically tailored to address ischemia. The therapeutic regimen includes T: botulinum toxin type A (BTX-A), H: high-dose HYAL, I: injectable platelet-rich fibrin (iPRF), S: serum platelet-rich fibrin (sPRF), a: aspirin and antibiotics, n: nanofat, d: debridement and dermabrasion, and F: fat membrane application.

**Results:**

A total of 25 eligible patients, including 20 women and 5 men with a mean age of 32.36 ± 6.71 years, were included. The THIS and FAT protocol involved the injection of BTX-A and HYAL, with mean doses of 50.68 ± 60.79 Units and 5970.0 ± 2791.65 IU, respectively. Additionally, iPRF and sPRF were applied to the ischemic wound surface. Debridement was performed for ischemia classified as stage three or higher. Notably, 92% of patients treated with the THIS and FAT protocol showed complete improvement without scar formation.

**Conclusion:**

“THIS and FAT” Protocol for managing ischemia following FIVO shows promising outcomes. Additionally, wound management with fat membrane, iPRF injections, sPRF dressing, and nanofat application resulted in favorable outcomes in this case series.

## Introduction

Hyaluronic acid (HA) has been a widely used biocompatible filler since the early 21st century, due to its safety and bio-compatibility. Despite its benefits, HA fillers can lead to serious complications such as infection, allergic reactions, and vascular occlusion, with the most severe being blood vessel blockage that can result in necrosis and scarring (1).

Prompt management of vascular occlusion, particularly within the first 72 h, is critical. High-dose perilesional hyaluronidase (HYAL) injections are recommended to create a concentration gradient with diffusion into vessels and facilitate the breakdown of the intravascular HA, causing vascular occlusion (2). Animal studies have shown that administering HYAL within 4 h of filler-induced vascular ischemia (FIVO) significantly improves affected areas (3).

Early intervention with HYAL, ideally within 48 h, is associated with better outcomes, while delays can lead to severe complications such as necrosis, scarring, blindness, and cerebrovascular accidents (4).

Key indicators of vascular occlusion include pain and acute skin discoloration, with persistent paleness being an early sign of ischemia (5). High-risk areas for occlusion include the glabella and nasolabial folds, with risk factors such as large volume injections, small sharp needles, deeper planes, and high-pressure injections (6). Several protocols for HYAL dosage and injection technique exist, but consensus favors high doses and timely administration for optimal efficacy (7). This study introduces a new protocol, “THIS and FAT,” designed to address ischemia after FIVO with a structured therapeutic approach and highlights the use of ultrasound-guided HYAL injections to overcome challenges related to varying filler depths and volumes (8). This study broadens a novel horizon for FIVO management, while the absence of a control group due to its emergency nature should be considered.

## Materials and methods

### Study design

This interventional study evaluates the efficacy of a novel protocol, termed “THIS and FAT,” for managing ischemia resulting from FIVO. The ethics committee of Fakih Hospital (Approval ID number 00001103) approved this study. The “THIS and FAT” protocol includes a therapeutic ladder comprising T: botulinum toxin type A (BTX-A), H: high-dose HYAL, I: injectable platelet-rich fibrin (iPRF), S: serum Platelet-Rich Fibrin (sPRF), a: aspirin, n: nanofat, d: debridement, and FAT: fat membrane. This investigation involved patients who experienced vascular occlusion at various stages of ischemia following HA filler injections and were referred to our private clinic between February 2023 and December 2024. All participants provided signed informed consent.

### Inclusion and exclusion criteria

Patients were included in the study if they exhibited symptoms and signs of ischemia following HA filler injections. Exclusion criteria comprised ischemia resulting from non-HA fillers, a history of hypersensitivity to HYAL, pregnancy, lactation, and ischemia affecting non-facial areas. Additionally, patients who had received an unspecified dose of hyaluronidase before referral were excluded. In total, 27 patients who met the eligibility criteria were included in the study, representing a spectrum of ischemia and necrosis stages.

### Pre-intervention evaluation

Demographic and clinical data were collected, including personal and medical history, time elapsed between filler injection and clinic visit, type and volume of injected filler, injection sites, and ischemia stage. Clinical stages of ischemia were assessed and classified by a head and neck surgeon in conjunction with two expert general physicians (9). Each patient underwent facial photography and an initial ultrasound examination.

### Ultrasound procedure

All patients underwent baseline portable ultrasonography prior to the initiation of any therapeutic interventions. The Clarius L20 handheld high-frequency ultrasound device (HD, 8–20 MHz) was utilized to identify the affected arteries within the injection sites. Following each hyaluronidase injection, a follow-up ultrasound examination was performed to monitor the restoration of normal arterial flow in the affected regions.

### Therapeutic Interventions: “THIS and FAT” protocol

1. BTX-A: As the initial therapeutic intervention, BTX-A (abobotulinum toxin A, Dysport^§,^ Galderma, Lausanne, Switzerland) is administered, with dosages ranging from 20 to 150 units depending on the specific area affected. Following this, the patient receives the first dose of HYAL.
2. HYAL: According to the Complications in Medical Aesthetics Collaborative (CMAC) protocol (9), which is a modification of the High Dose Pulse Hyaluronidase (HDPH) protocol (2), we administer 1,500 units of HYAL every 20 min for each angiosome. Each 1,500-unit vial of HYAL is mixed with 1 cc of 2% lidocaine and injected into the ischemic areas. HYAL is delivered perivascularly in each ischemic zone, with efforts made to inject an intra-arterial bolus under ultrasound guidance. This approach utilizes both the diffusion effect of HYAL for filler dissolution and the precise intra-arterial administration to eliminate HA. The HYAL injections continue until both clinical capillary refill time (CRT) and ultrasound assessments of arterial flow return to normal.
3. iPRF: Two polyethylene terephthalate (PET) centrifuge tubes, each containing 10 mL of whole blood without anticoagulant, are used. Following established protocols (10, 11), the PET tubes are centrifuged at 4,000 rpm for 2 min at room temperature. The resulting iPRF is then applied to the wound surface.
4. sPRF: Under the same conditions, PET tubes are centrifuged at 3,500 rpm for 3 min to obtain sPRF. This serum covers the wound surface of all ischemic skin lesions categorized as stage two or higher.
5. Debridement and dermabrasion: In cases of ischemia classified as stage three or higher, debridement of the wound surface is performed and then finished with a soft dermabrasion.
6. Nanofat: Fat harvesting is conducted under local anesthesia, using a modified Klein solution consisting of 500 mL normal saline, 10 mL 2% lidocaine, and 1 mL of 1:1,000 adrenaline. Fat is harvested using a microfat cannula attached to a 10-mL Luer Lock syringe. The aspiration is carried out under low negative pressure. For the preparation of Nanofat, the microfat is emulsified through three successive filters (2.4, 1.4, and 1.2 mm), with the process involving approximately 30 passes between two 20-mL syringes for each filter. The resulting Nanofat is then applied to the wound surface and injected into the ischemic area using the Sharp-Needle Intradermal Fat Grafting (SNIF) technique (12).
7. Fat Membrane: Finally, the fat membrane (nanofat or microfat membrane) is employed as an effective treatment for repairing ischemic wounds. It is carefully sutured over the ischemic area, as outlined by the authors in a previous published study (13).

**FIGURE 1:**
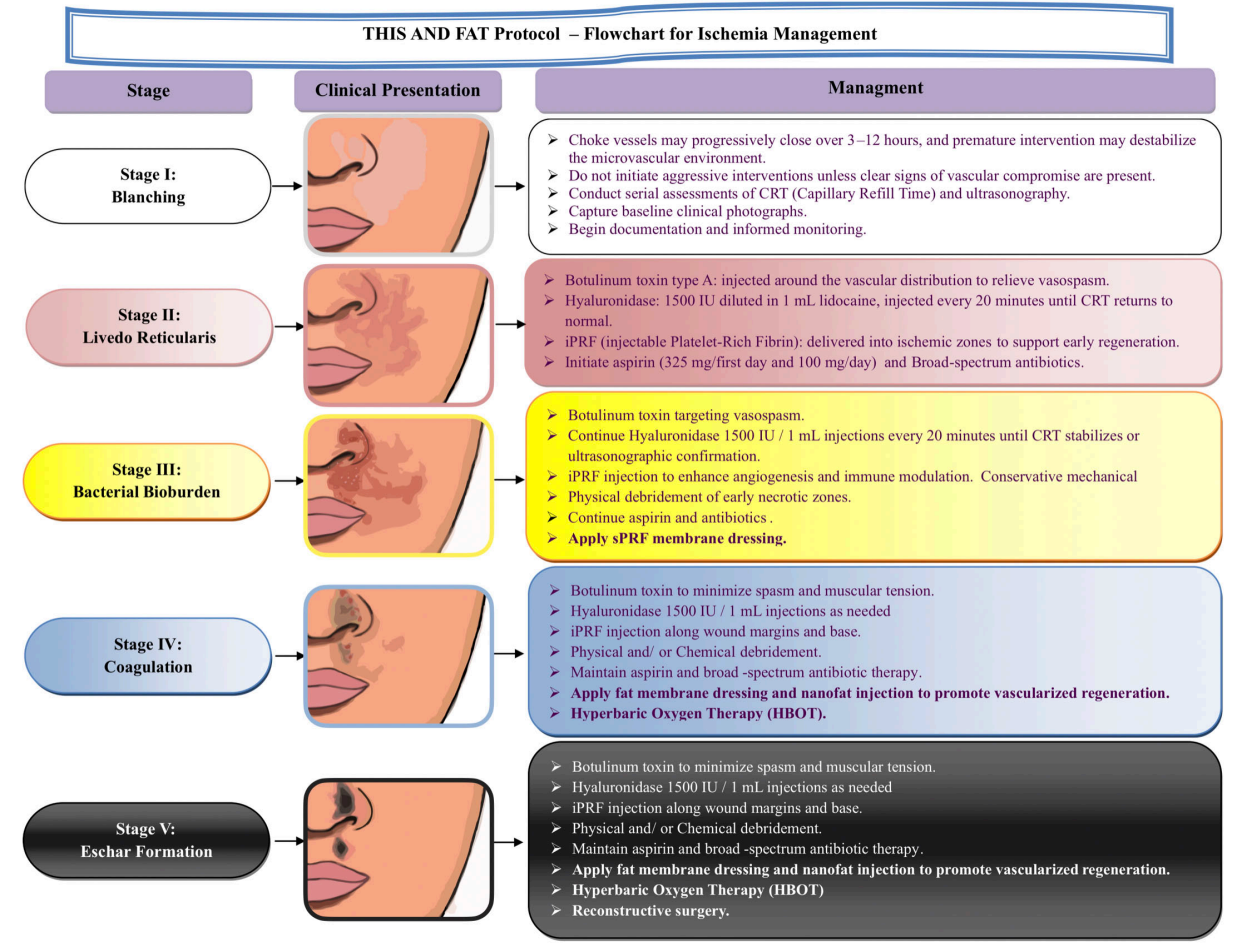
Flowchart for ischemia management: the THIS and FAT protocol. This staged management protocol outlines the clinical presentation and step-by-step treatment strategy for vascular compromise following dermal filler injections. The stages progress from early blanching (Stage I) to eschar formation (Stage V). At each stage, specific interventions are recommended, including serial assessments of capillary refill time (CRT), the use of botulinum toxin to relieve vasospasm, hyaluronidase injections, injectable platelet-rich fibrin (iPRF), selective antibiotic therapy, and advanced wound care techniques. In more advanced stages (Stages IV and V), additional therapies such as fat membrane dressing, nanofat injection, hyperbaric oxygen therapy (HBOT), and reconstructive surgery are introduced to promote tissue regeneration and recovery. This protocol aims to provide a structured and dynamic approach to managing filler-induced vascular occlusion (FIVO).

A flowchart-based algorithm was developed to guide ischemia management according to five clinical stages, outlining corresponding step-by-step interventions from early detection to advanced tissue regeneration (Figure 1).

### Post-treatment evaluations

Following treatment, CRT and ultrasound examinations are documented for each patient. A standard regimen of aspirin 100 mg daily, Pentoxifylline 400 mg three times daily (TDS), Clindamycin 150 mg TDS, and Levofloxacin 750 mg daily for 7–10 days is prescribed to all patients (14). Follow-up visits are scheduled for days 1, 3, 7, and 30 post-treatment. Additional follow-up visits are conducted for some patients at 3, 6, and 12 months. Depending on the stage of ischemia and clinical evaluation, patients must submit facial photographs every 1–2 days, and follow-up imaging is performed during each visit. Monitoring continues through photographic and clinical evaluations until complete resolution of facial skin issues or the formation of scarring is achieved.

### Statistical method

The continuous variables were statistically described as Mean ± SD. Moreover, the frequency and percentage of categorical variables were reported. The Chi-square was employed to evaluate the association between categorical variables. A *p*-value of less than 0.05 is considered statistically significant. Statistical analyses are conducted using SPSS software version 26.

## Results

Of the 27 patients initially enrolled in the study, two were excluded due to the use of alternative therapeutic protocols. The demographic, clinical characteristics, and complication-related data for the remaining participants are detailed in Table 1. Thus, the study included 25 patients—20 women and 5 men—with a mean age of 32.36 ± 6.71 years. All patients had undergone HA filler injections: 10 patients received 10 cc of body filler, and 15 patients received 1 cc of standard facial filler. Due to the high cost and rising popularity of cosmetic procedures, some practitioners use 10 cc body fillers for facial applications. The most frequent injection sites leading to vascular occlusion were the nose (*n* = 9), temple (*n* = 4), chin (*n* = 4), glabella (*n* = 3), lips (*n* = 3), and nasolabial folds (*n* = 2).

**TABLE 1:** The demographic data of each patient with ischemia.

N	Sex	Age	Type of filler	Volume	Injection site	Needle or cannula	Sign and symptom	Stage of ischemia	Area (CM^2^)	Previous rhinoplasty	Smoking	Treatment plan	Final result
1	F	17	Dene-B	1 cc	Nose tip	Needle	-Pain-Blanching	IV-V 144 h		No	No	-Hyaluronidase 12,000 U -iPRF -SPRF dressing −300 U Abo BoNT-A -Fat membrane	The support layer remained intact −4CM^2^ scar formation
2	M	28	Perfectha	0.3 cc	Glabella	Needle	-Tenderness	III 84 hours	10.5	No	No	-Hyaluronidase 5,500 U -iPRF -sPRF dressing −150 U Abo BoNT-A	No scar
3	F	34	EPTQ S500	1 cc	Temple	Cannula 22G	Headache Tenderness Erythema	III-IV 90H	40	Yes	No	-Hyaluronidase 9,000 U −20 U Abo BoNT-A -iPRF -sPRF	Hair loss in the temple area and hair regrowth after 4 months without any scar
4	F	38	Zishel 10 cc	1 cc	NLF	Needle	-Pain -Tenderness -Erythema and LR	III 80H		No	No	-Hyaluronidase 2250 U -iPRF -SPRF dressing −100 U Abo BoNT-A	No scar
5	M	26	Audrey 10 cc	0.3 cc	Temple	21G Cannula	-Pain -Tenderness - LR	III 52H	50	Yes	Yes	-Hyaluronidase 6,000 U -iPRF -SPRF dressing −40 IU Abo BoNT-A	No scar
6	F	38	Dermofill 10 cc	2 cc	Lower lip	Needle	-Pain -Tenderness - LR -Eschar formation and necrosis	IV-V 137H	5.25	Yes	No	−50 IU BoNT-A injection -Hyaluronidase 6,000 U -iPRF injection -SPRF dressing -Nanofat and SVF gel and microfat injection	Scar formation (1 CM^2^)
7	M	35	Neuvia intense	0.1 cc	dorsum	Needle	-Pain -Tenderness - LR	II 50H	4.5	No	Yes	−20 IU Abo BoNT-A −3,000 IU Hyaluronidase	No scar
8	F	40	DeneB 10 cc	1 cc	NLF with Marionette Involvement of nasal cavity and oral cavity	Needle	-Pain -Tenderness -edema -Bacterial bioburden	III 74H	40	Yes	No	−50 IU BoNT-A injection -Hyaluronidase 9,000 IU -iPRF injection -SPRF dressing	No scar
9	F	32	Neuramis	0.5 cc	Temple	Cannula 23G	-Pain -Tenderness - LR	II 78H	24	No	No	30 IU Abo BoNt-A −9000 IU Hyaluronidase iPRF	No scar
10	F	43	Fillarmony	0.5 cc	Dorsum of the Nose, Nasal cavity	Needle	-Pain -LR -Tenderness - periorbital edema	III 77H	8.5	No	No	−100 IU Abo BoNt-A −7500 IU Hyaluronidase -iPRF	No scar
11	M	21	Neuramis	0.4 cc	Glabella	Needle	-Pain -LR	II 64H	36	No	No	−50 IU BoNT-A Abo −6,000 IU Hyaluronidase -iPRF	No scar
12	F	23	Neuramis	1.8 cc	Lips	Needle	-Pain - LR	II 16H	5.4	No	No	−30IU Abo BoNT-A −3,000 IU Hyaluronidase	No scar
13	F	27	Neuramis	0.3 cc	Dorsum	Needle	-Pain - LR -Edema	II 45H	6	No	No	−30 IU Abo BoNT-A −4,500 UI Hyaluronidase	No scar
14	F	32	Neuramis	1 cc	Alar rim and Nose tip	Needle	-Pain -LR -Eschar formation	IV-V 174H	2	Yes	No	−30 IU abo BoNT-A −6,000 IU Hyaluronidase Four times dermabrasion and iPRF injection, and sPRF dressing	No scar
15	F	33	Audrey 10 cc	0.5 cc	Nose tip	Needle	-Pain - LR -Bacterial bioburden	III 48H	4.5	Yes 2times	Yes	−30 IU Abo BoNT-A −6,000 IU Hyaluronidase -iPRF injection -sPRF dressing	No scar
16	F	31	Inovosense	0.5 cc	Temple	Cannula 18G	-Pain - LR	II 119H	53	No	No	−20 IU Abo BoNT-A -6,000 IU Hyaluronidase -iPRF injection -sPRF dressing	No scar
17	F	31	Revofil ultra 1 cc	0.5 cc	Chin	Needle	- LR	II 148H	3.5	Yes	No	−30 IU Abo BoNT-A −3,000 IU Hyaluronidase -iPRF injection -sPRF dressing	No scar
18	M	38	Zishel 10 cc	0.3 cc	Nose	Needle	-Pain - LR -Epistaxis -Bacterial bioburden	IV 94H	4	Yes	Yes	−30 IU Abo BoNT-A −4,500 IU Hyaluronidase -iPRF injection -sPRF dressing - fat membrane dressing	No scar
19	F	28	Nuxiwell 1 cc	1 cc	Upper lip	Needle	-Pain - LR	III-IV 77H	3.5	No	Yes	−14 IU Abo BoNT-A −3,500 IU Hyaluronidase -iPRF injection -sPRF dressing	No scar
20	F	31	Revofill ultra 1.0 cc	0.5 cc	Chin	Needle	-Pain - LR	II 147H	3.5	Yes	No	-30 IU Abo BoNT-A −3,000 IU Hyaluronidase -iPRF injection -sPRF dressing	No scar
21	F	42	Dene-B	1 cc	Chin	Needle	-Pain - LR -Bacterial bioburden	III 87H	12	No	No	−20 IU Abo BoNT-A −13,500 IU Hyaluronidase -iPRF injection -sPRF dressing	No scar
22	F	28	Revofil 1.0 cc	0.3 cc	Nose	Needle	-Pain - LR	II 28H	10	Yes	No	-30 IU Abo BoNT-A −4,500 IU Hyaluronidase -iPRF injection -sPRF dressing	No scar
23	F	42	Zishel 10 cc	0.7 cc	Chin	Needle	-Pain - LR -Bacterial bioburden	III 66H	4	No	No	−15 IU Abo BoNT-A −6,000 IU Hyaluronidase -iPRF injection -sPRF dressing	No scar
24	F	33	Neuramis	0.1 cc	Nose Soft triangle	Needle	-Pain - LR -Bacterial bioburden	III 72H	6	Yes Two times	No	-28IU Abo BoNT-A -6,000 IU Hyaluronidase -iPRF -sPRF	No scar
25	F	38	EPTQ S500	0.15 cc	Glabella	Needle	-Blurred vision -Paleness -LR	II 10:30H	44	No	No	−20 IU Abo BoNT-A −4,500 IU Hyaluronidase -iPRF	No scar

F, Female; LR, Livedo reticularis; M, Male; N, Number, Abo-BoNT-A (AbobotulinumtoxinA).

Patients experienced ischemic events after varying volumes of filler injection, with a mean volume of 0.67 ± 0.48 cc (range: 0.1–2.0 cc). The average time between filler injection and referral to our clinic for ischemia management was 82.48 ± 42.37 h. Our clinic, specializing in ischemia after FIVO, serves as a referral center for delayed vascular complications.

The mean area affected by ischemia was 16.53 ± 17.45 cm^2^, ranging from 2 to 53 cm^2^. Notably, 84% of vascular occlusions were associated with needle injections in various facial areas, while 16% of complications arose from cannula injections, all occurring in the temple.

Pain was the most common symptom following vascular occlusion, reported by 92% of patients. The predominant signs included livedo reticularis in 84% of patients and bacterial bioburden in 24%. The patients presented with varying stages of ischemia: stage two (40%, *n* = 10), stage three (36%, *n* = 9), stage four (12%, *n* = 3), and stage five (12%, *n* = 3). The highest stage of ischemia was recorded for each patient.

The therapeutic management of ischemia employed the “THIS and FAT” protocol, an acronym designed to facilitate the application of the ischemia treatment protocol. The protocol includes BTX-A, HYAL, iPRF, sPRF, aspirin, nanofat, debridement, dermabrasion, and Fat membrane. This structured approach helps physicians and injectors manage vascular occlusions effectively.

In the “THIS and FAT” protocol, BTX-A was administered as the initial intervention, with a mean dose of 50.68 ± 60.79 units (range: 14–300 units). HYAL was then administered at a mean dosage of 5970.0 ± 2791.65 IU. iPRF and sPRF were applied to the ischemic wound surfaces for all stage two or higher ischemia cases. Additionally, debridement was performed for all patients with stage three or higher ischemia to minimize bacterial load. iPRF and sPRF were used in 88 and 72% of patients, respectively. For advanced ischemia or resistant wounds, the fat membrane (nanofat or microfat membrane) was utilized to enhance wound healing; this treatment was applied to three patients with stage four and up.

No significant association was found between a history of rhinoplasty and an increased risk of vascular occlusion in the nose or nasolabial folds compared to other areas. Among the patients with advanced ischemia (stages 4 and 5), two developed scar formation, a significantly higher rate compared to those with lower stages of ischemia (*P* = 0.009). Additionally, patients with advanced ischemia had a significantly longer referral time (119.33 ± 37.96 h) compared to those with less severe ischemia (70.84 ± 37.39 h, *P* = 0.01). The volume of filler leading to advanced ischemia was also higher (1.05 ± 0.54 cc) compared to stages 1, 2, and 3 (0.55 ± 0.41 cc, *P* = 0.03). The amounts of Botox and hyaluronidase used did not significantly differ between advanced and less severe stages of ischemia.

**FIGURE 2:**
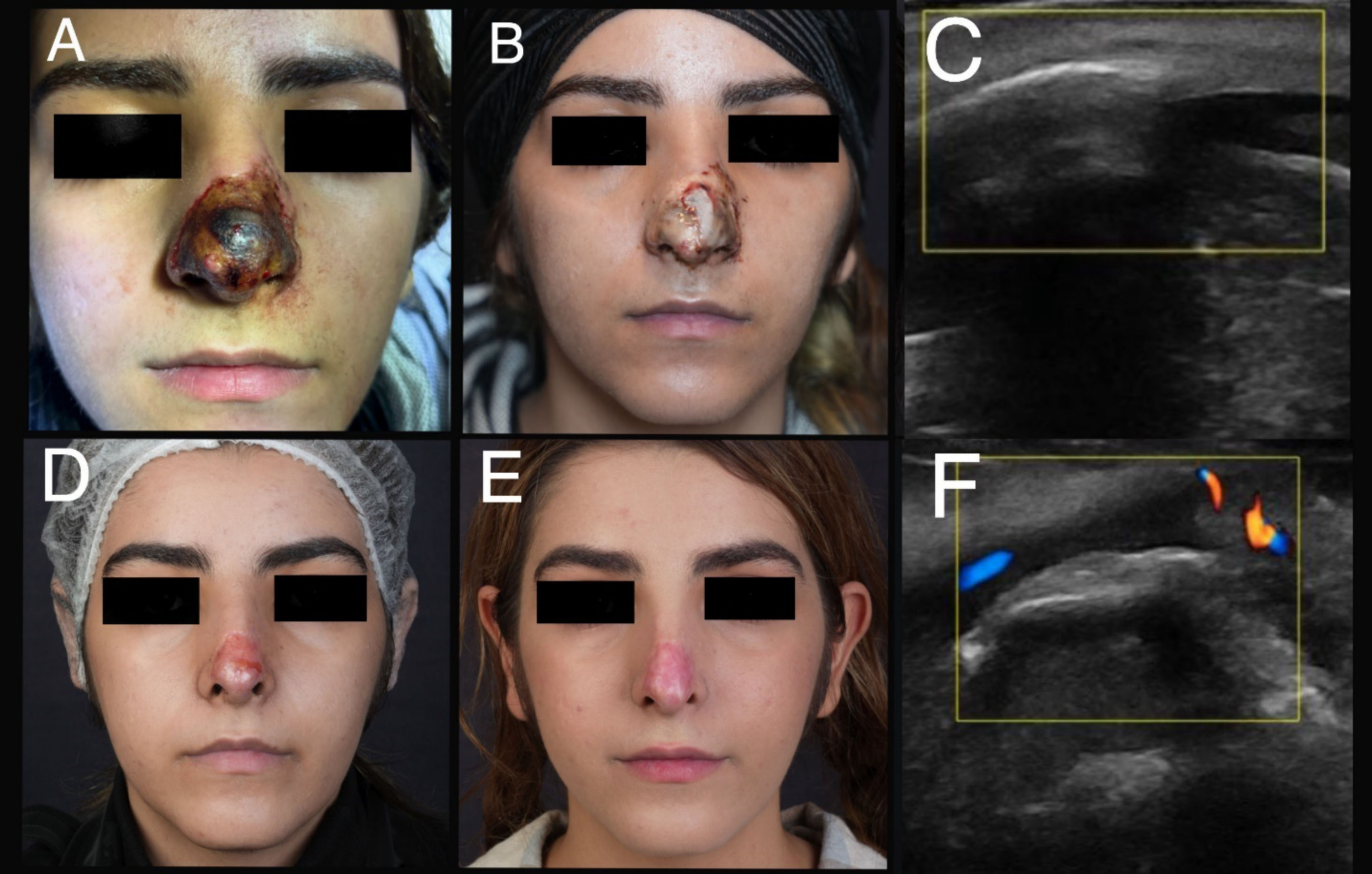
A 17-year-old female experienced stage five of ischemia following an injection of 1 cc HA filler. **(A)** A clinical presentation after 144 h of filler injection. **(B)** A day after treatment with “THIS and FAT” protocol. **(C)** An ultrasound examination before treatment (Involvement of lateral nasal artery). **(D)** Improvement of the lesion after 3 months. **(E)** Atrophic scar after 8 months of filler injection. **(F)** An ultrasound examination after treatment (arterial flow return to normal).

**FIGURE 3:**
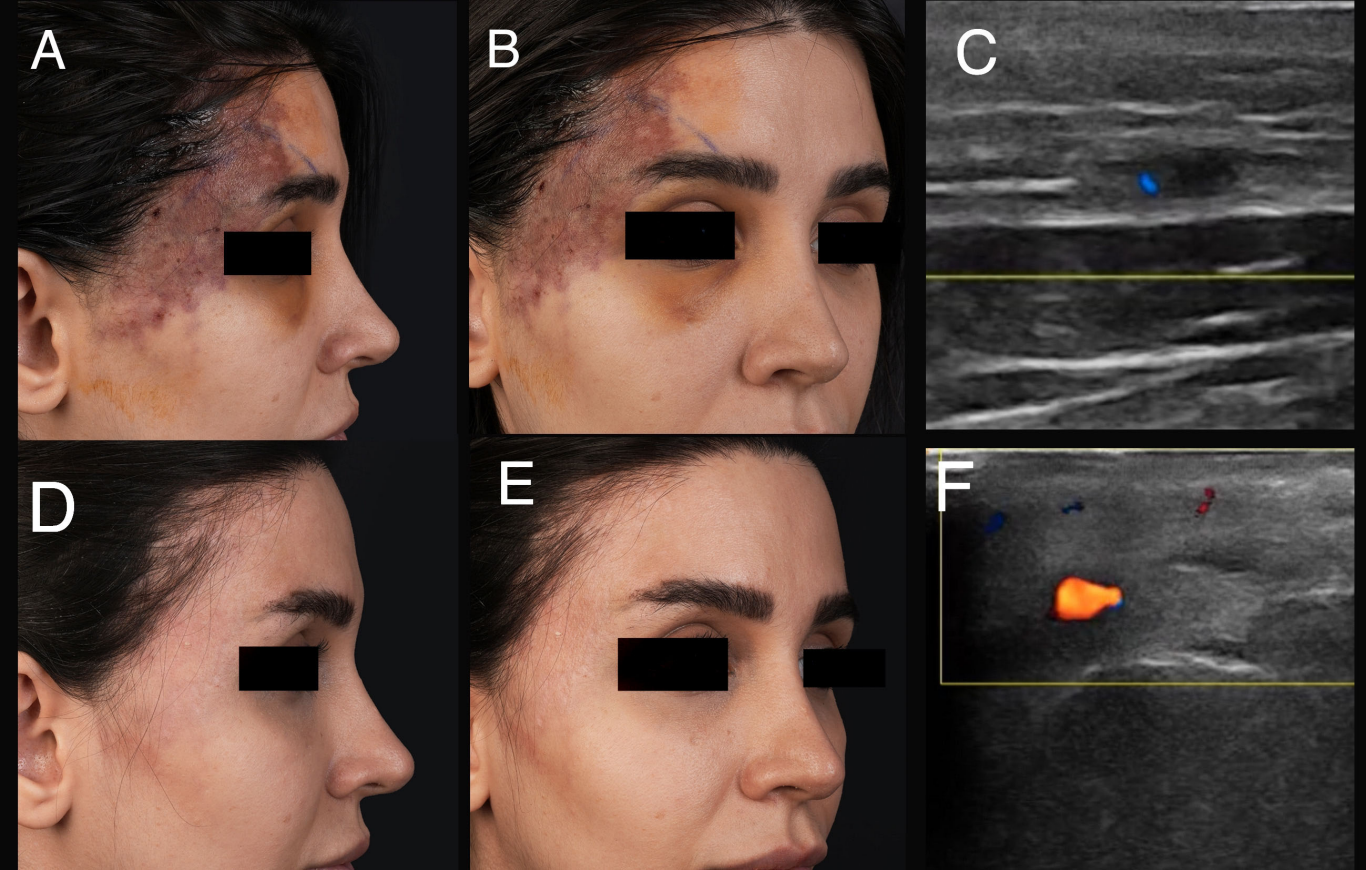
A 31-year-old female experienced ischemia following an injection of 0.5 cc HA filler. **(A,B)** A clinical presentation after 119 h of filler injection. **(C)** An ultrasound examination before treatment (Involvement of frontal branch of superficial temporal artery). **(D,E)** Improvement of ischemia a month after the “THIS and FAT” protocol. **(F)** An ultrasound examination after treatment (arterial flow return to normal).

**FIGURE 4:**
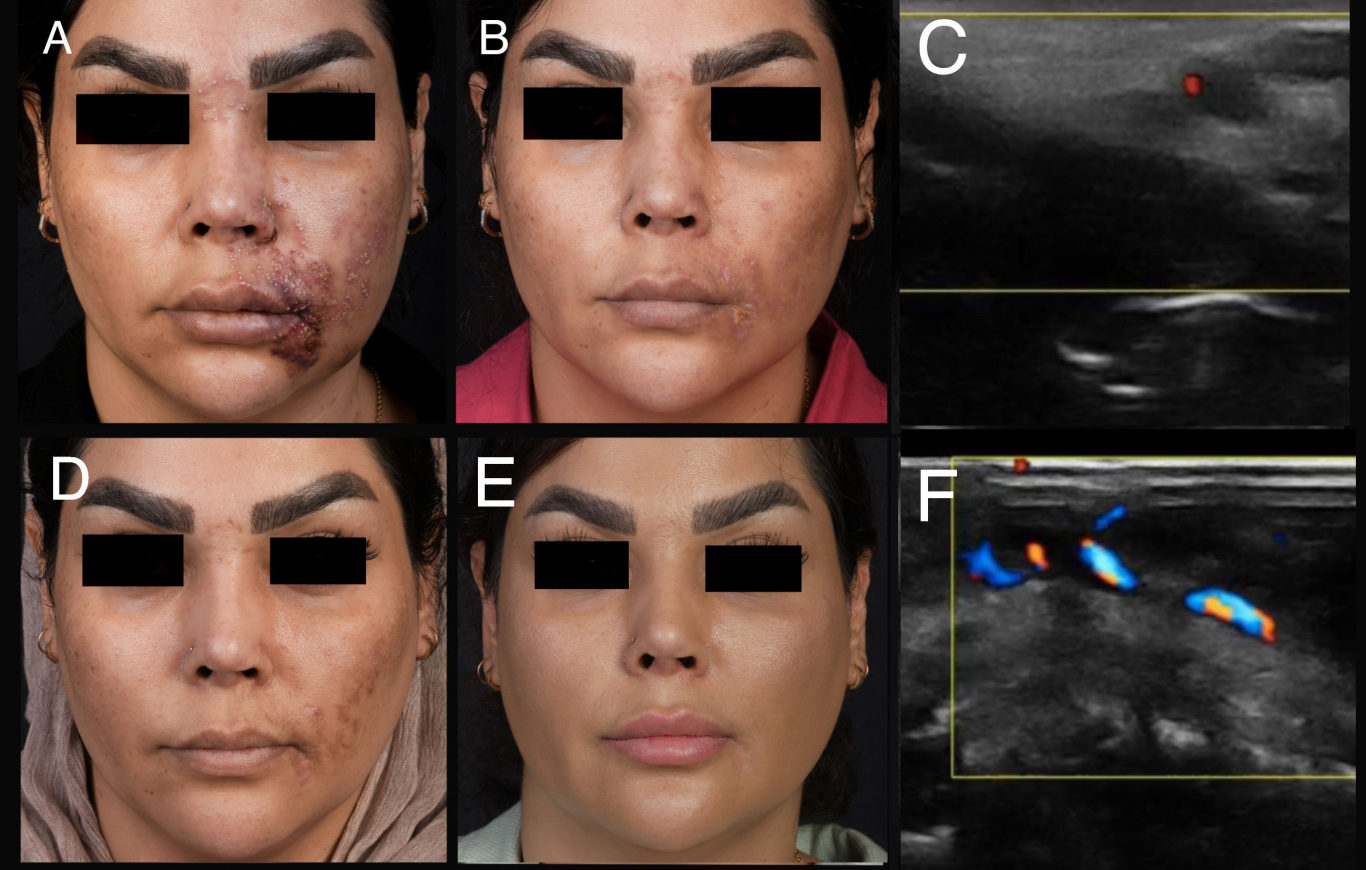
A 40-year-old female experienced stage three of ischemia following an injection of 1 cc HA filler. **(A)** A clinical presentation after 74 h of filler injection. **(B)** Improvement of ischemia after 2 weeks. **(C)** An ultrasound examination before treatment (Involvement of facial artery). **(D)** Improvement of ischemia a month after filler injection. **(E)** Complete improvement 3 months after “THIS and FAT” protocol. **(F)** An ultrasound examination after treatment (arterial flow return to normal).

Only two patients with advanced ischemia or presenting more than 72 h after the occlusion developed scar formation (Figure 2). Consequently, 92% of patients treated with the “THIS and FAT” protocol achieved complete recovery without scarring (Figures 3, 4).

The main side effect observed was facial unilateral asymmetry due to BTX-A injection. This complication should be noted in the nasolabial fold or cheek ischemia. We recommend administering BTX-A on the contralateral side to achieve acceptable cosmetic results.

## Discussion

The incidence rate of ischemic events following vascular compromise is approximately 3 in 1,000 injections (15, 16). Ischemia after filler injection can progress through five clinical stages: Stage I presents immediately with skin blanching and delayed capillary refill; Stage II may develop rapidly and persist for over 48 h, characterized by livedoid skin changes due to the pooling of deoxygenated blood in under-perfused regions; Stage III typically occurs around 72 h post-injection and is marked by the deterioration of the skin barrier, partial desquamation, and overgrowth of cutaneous flora; Stage IV manifests 5–10 days post-injury, featuring coagulative necrosis, which can eventually lead to the formation of an eschar (Stage V) that may persist for weeks (9). Based on our experience, some patients presented with Stage III ischemia as early as 24 h after injection, while others experienced advanced ischemia 48–72 h post-injection, suggesting that the timeline for the clinical stages of ischemia may vary. Treatment for patients in Stages I and II is most effective when following the HDPH protocol, which recommends increasing the HYAL dosage in affected areas (17). Early palliative therapies should also be considered, including local dressing, acetylsalicylic acid, non-steroidal anti-inflammatory medication, and massage (18).

Several protocols have been proposed to improve the management of vascular occlusion. For example, Lee et al. reported more favorable outcomes with a protocol involving the injection of 125 units of HYAL every 15 min, compared to a single bolus dose of 500 units (19). In this protocol, 77% of patients with ischemia showed significant recovery, while only 49% experienced complete resolution without scarring (19). In the “THIS and FAT” protocol, which was applied to several patients with advanced ischemia, 92% of cases improved completely without any scar formation.

Managing ischemia after the third stage becomes increasingly challenging, as it requires not only addressing the occlusion but also managing wound care. For wound care, the application of various growth factors, including Platelet-Rich Plasma (PRP) and stem cells, is recommended (20). Incorporating these adjunctive therapeutic modalities into a comprehensive protocol helps physicians manage skin necrosis more effectively. However, HYAL injections can be complicated by the presence of infection, as increased tissue permeability raises the risk of spreading the infection.

In this case series, we presented a novel protocol for managing ischemia following HA injection. In the first stage, all patients were treated with BTX-A, using a dosage of 20–150 units for multiple purposes. During ischemia caused by filler injection, the choke vessels in the tissue undergo vasoconstriction to limit ischemia. While this vasoconstriction may be beneficial during the first 6 h, which constitutes the acute phase of ischemia, it can exacerbate the condition beyond 6 h by impeding tissue repair (21). Additionally, lactic acid is produced during ischemia due to anaerobic respiration, leading to muscle contraction and further worsening of the ischemic condition (22).

BTX-A injections aimed at inducing vasodilation have been extensively studied in animal models, demonstrating enhanced blood flow and improved outcomes in ischemia (23, 24). Additionally, several studies have reported the efficacy of BTX-A in treating various ischemia-related conditions. In cases of Raynaud’s phenomenon (RP), BTX-A has shown beneficial effects, such as increased digital temperature, improved blood flow and oxygen saturation, and a general reduction in RP symptoms (25). Notably, BTX-A has demonstrated positive vasodilation effects in patients within 30 min of administration (26), likely by blocking sympathetic nerve conduction, which reduces vasoconstriction (25, 27). Moreover, BTX-A has been shown to increase endothelial nitric oxide synthase (eNOS) activity and elevate cGMP levels in human microvascular endothelial cells, leading to reduced contraction of endothelium-intact arteries. These findings support the vasodilatory effects of BTX-A injections (27). Another study evaluated the vascular effects of BTX-A on human skin by injecting it subcutaneously into low-perfusion areas of 40 patients’ backs. Results showed that BTX-A significantly increased blood vessel diameter (up to 2.4×), blood flow (up to 5.5×), and led to the emergence of new perforating vessels. These changes were confirmed through Doppler ultrasound and thermography, which also detected a 2–5°C local temperature rise. The control group (NaCl 0.9%) showed no vascular changes. The findings highlight BTX-A’s potential to improve skin perfusion and stimulate neovascularization in compromised tissues (28).

In our “THIS and FAT” protocol, we begin by injecting BTX-A, and ultrasound examinations have shown substantial vasodilation of arteries following BTX-A injections. However, to date, no studies have specifically investigated the efficacy of BTX-A in managing ischemia. Furthermore, Goldberg et al. reported treating 20 patients with ischemic vasospasm using BTX-A, which resulted in rapid and sustained pain relief lasting several months (29).

Based on the authors’ experience, BTX-A injections can be beneficial in ischemic conditions that have progressed beyond the acute stage (after 6 h) (26). The primary complication associated with BTX-A injections is unilateral asymmetry. In certain areas—such as the glabella, chin, and nose—this asymmetry is often clinically insignificant, whereas in regions like the nasolabial fold and cheek, injecting the contralateral side can enhance aesthetic outcomes. To minimize this risk, we inform all patients of the potential for asymmetry and recommend treating both sides to achieve better cosmetic results. Additionally, no other adverse events related to BTX-A administration were observed in our patients.

Following the administration of BTX-A, HYAL injection is used as the second stage of treatment. HYAL, an enzyme commonly utilized in medicine, enhances tissue permeability. Its application has shown positive effects on ECG outcomes in cases of cardiac necrosis (30). In 2014, Claudio de Lorenzi demonstrated that HYAL can permeate the vessel wall and enter vessels through concentration gradient and diffusion mechanisms (31). Furthermore, de Lorenzi introduced the HDPH protocol in 2017 to effectively manage vascular occlusion, recommending a minimum of 500 IU of HYAL for every 3 × 3 cm area (2). The HYAL injection should be initiated in the periphery of ischemic areas and subsequently injected into the nearest point to vascular occlusion due to the possibility of HA fragment embolization. HA embolization may lead to other complications, such as blindness.

Subsequently, the CMAC modified de Lorenzi’s protocol, taking into account the tissue half-life of HYAL, which is reported to be 5.1 min subcutaneously and 7.5 min intramuscularly (9). In cases of ischemia caused by filler injections, HYAL should be diluted in lidocaine or normal saline at a concentration of 1,500 units per 1–2 cc. The standard approach is to administer 1,500 units every 20 min (9).

Authors recommend diluting 1,500 units of HYAL in 1 cc of lidocaine and administering it at 20-min intervals, considering the increased concentration gradient and potency. Plain 1 and 2% lidocaine have a pH of 6.09 ± 0.16 and 6.00 ± 0.27, respectively (32). Also, regarding HYAL type, it is activated in range of PH from 3 to 8, and its combination with plain lidocaine provide optimal PH activity (33). HYAL injections should continue until improvement in arterial flow is confirmed via ultrasound and CRT. Also, the “THIS and FAT” protocol emphasizes intra-arterial HYAL injection under the guide of sonography (IS: intra-arterial injection under sonography). Several studies report increased efficacy of intra-arterial HYAL injection with lower dosages (34). Regarding our experience, intra-arterial HYAL administration under ultrasound guidance leads to favorable outcomes. Interestingly, Zhang et al. reported a decrease in pure crosslinked HA particle size from 400 to 600 μm to 300 to 400 μm following continuous hydrolysis by HYAL (35).

Notably, fillers with high viscosity, such as HA may associated with more vascular obstruction, while fillers with low viscosity do not cause complete vascular occlusion (36). Fillers with low viscosity may result in more embolism, increasing the risk of secondary vascular occlusion following HYAL administration. However, regarding the few reports of viscosity in FIVO cases, the assessment of different cross-linking filler effects for vascular complications is limited (37). Several HYAL-related complications have been reported, including local allergic reactions, urticaria, angioedema, as well as immediate and delayed hypersensitivity reactions, particularly at doses exceeding 100,000 IU. In our cases, no adverse events related to HYAL administration were observed.

The next step involves wound management, which includes the injection of iPRF, application of sPRF dressing, nanofat injection, or the use of Stromal Vascular Fraction (SVF) gel. PRF plays a significant role in treating necrosis due to its angiogenic factors, such as platelet-derived growth factor (PDGF), transforming growth factor beta (TGF-β), epidermal growth factor (EGF), vascular endothelial growth factor (VEGF), insulin-like growth factor I (IGF-I), platelet-derived epidermal growth factor (PDEGF), and platelet factor 4 (PF-4) (38). The fibrin matrix in PRF allows for the slow release of these factors, enhancing PRF’s therapeutic effects. Additionally, PRF has been reported to have antibacterial properties, and iPRF can increase tissue permeability and rehydrate ischemic tissue (39–41).

Given the imbalance of electrolytes, decreased tissue oxygenation, and the disproportion of various cytokines and antibodies, using iPRF helps improve skin viability by addressing these issues (42, 43). PRF has a high concentration of blood proteins, including protease inhibitors, complement proteins, and immunoglobulin G. Additionally, increased amounts of the mentioned proteins, as well as albumin, haptoglobin, and fetuin-A, contribute to wound healing (44). We recommend iPRF injections in all cases of ischemia following filler injection, especially in Stage 2 or higher. Several complications have been reported following PRP injections, including infection, inflammatory reactions, allergic reactions, nodular formation, and even blindness. In contrast, PRF application has not been associated with any serious or major complications (45, 46).

When a patient with ischemia presents, particularly in the presence of bacterial bioburden (Stage 3 or higher), the primary step is to debride infected and necrotic tissues to prevent the spread of infection through hyaluronidase injections. Surgical debridement is essential for preparing the wound bed in necrosis (47). The rationale for early debridement is to remove the source of infection from the ischemic site and to conserve ATP by preventing the consumption of energy on dead cells within the necrotic tissue (48).

In patients with Stage 3 or higher ischemia, we prioritize debridement of the affected areas, followed by the administration of BTX-A and HYAL. Nanofat and SVF gel, which contain adipose-derived stem cells (ADSC), play a crucial role in tissue regeneration within necrotic wounds. Nanofat, in particular, has unique angiogenic properties due to growth factors like VEGF, PDGF, and FGFb (49–51). Additionally, nanofat grafting promotes neocollagenesis, tissue regeneration, and skin rejuvenation. ADSC also contributes significantly to skin rejuvenation and the inhibition of melanocyte proliferation (52). While several complications have been reported with macrofat or microfat, nanofat administration is considered safe, with only transient and reversible adverse events such as bruising or erythema (53).

Given these characteristics, the combination of iPRF, sPRF, and Nanofat significantly enhances the regeneration of necrotic tissue. To further promote tissue repair in stages 4 and 5, we apply a fat membrane (nanofat membrane) to the wound surface in ischemic regions following soft dermabrasion. This method leverages the fat membrane’s notable regenerative properties and has demonstrated significant effectiveness in tissue repair, particularly in cases of ischemia. Our previous research has thoroughly detailed the methodology and efficacy of using a fat membrane for tissue regeneration (13). The authors recommend using fat membrane for all Stage 4 and 5 ischemia cases, as their clinical experience demonstrates superior outcomes compared to previous treatments.

The main limitation of this study was the absence of a control group. Due to the emergency nature of FIVO, the inclusion of a control group was not feasible. In urgent conditions like ischemia and necrosis, the best possible therapeutic management should be applied, which led us to treat all patients using our exclusive “THIS and FAT” protocol to achieve favorable outcomes.

For future studies, it is recommended to design a study that determines the optimal injection route (local or intra-arterial) and further explores the broader applications of the “THIS and FAT” protocol in the management of skin necrosis.

## Conclusion

Ischemia following HA filler injection is a rare but serious complication. The “THIS and FAT” protocol integrates several therapeutic approaches to treat ischemia and necrosis. Notably, most cases (92%) with treatment of “THIS and FAT” protocol showed complete improvement without scarring. Also, further studies are recommended to evaluate the efficacy of this protocol.

## Data Availability

The raw data supporting the conclusions of this article will be made available by the author’s, without undue reservation.

## References

[1] Choi S Shin S Seok J Yoo K Kim B. Management strategies for vascular complications in hyaluronic acid filler injections: a case series analysis. *J Cosmet Dermatol.* (2023) 22:3261–7. 10.1111/jocd.15990 37694495

[2] DeLorenzi C. New high dose pulsed hyaluronidase protocol for hyaluronic acid filler vascular adverse events. *Aesthet Surg J.* (2017) 37:814–25. 10.1093/asj/sjw251 28333326

[3] Li J Xu Y Wang Y Hsu Y Wang P Li J. The role of hyaluronidase for the skin necrosis caused by hyaluronic acid injection-induced embolism: a rabbit auricular model study. *Aesthetic Plast Surg.* (2019) 43:1362–70. 10.1007/s00266-019-01398-2 31139914

[4] Borzabadi-Farahani A Mosahebi A Zargaran DA. Scoping review of hyaluronidase use in managing the complications of aesthetic interventions. *Aesthetic Plast Surg.* (2024) 48:1193–209. 10.1007/s00266-022-03207-9 36536092 PMC10999391

[5] Loh K Phoon Y Phua V Kapoor K. Successfully managing impending skin necrosis following hyaluronic acid filler injection, using high-dose pulsed hyaluronidase. *Plast Reconstr Surg Glob Open.* (2018) 6:e1639. 10.1097/GOX.0000000000001639 29616162 PMC5865919

[6] Chiang Y Pierone G Al-Niaimi F. Dermal fillers: pathophysiology, prevention and treatment of complications. *J Eur Acad Dermatol Venereol.* (2017) 31:405–13. 10.1111/jdv.13977 27662522

[7] Zheng C Fu Q Zhou G Lai L Zhang L Zhang D Efficacy of percutaneous intraarterial facial/supratrochlear arterial hyaluronidase injection for treatment of vascular embolism resulting from hyaluronic acid filler cosmetic injection. *Aesthet Surg J.* (2022) 42:649–55. 10.1093/asj/sjab425 34958671

[8] Munia MA Munia CG Parada MB Ben-Hurferraz Parente J Wolosker N. Doppler ultrasound in the management of vascular complications associated with hyaluronic acid dermal fillers. *J Clin Aesthet Dermatol.* (2022) 15:40–3. PMC8884183 35309877

[9] Murray G Convery C Walker L Davies E. Guideline for the management of hyaluronic acid filler-induced vascular occlusion. *J Clin Aesthet Dermatol.* (2021) 14:E61–9. PMC8211329 34188752

[10] Karde P Sethi K Mahale S Khedkar S Patil A Joshi C. Comparative evaluation of platelet count and antimicrobial efficacy of injectable platelet-rich fibrin with other platelet concentrates: an in vitro study. *J Indian Soc Periodontol.* (2017) 21:97–101. 10.4103/jisp.jisp_201_17 29398852 PMC5771122

[11] Miron R Chai J Fujioka-Kobayashi M Sculean A Zhang Y. Evaluation of 24 protocols for the production of platelet-rich fibrin. *BMC Oral Health.* (2020) 20:310. 10.1186/s12903-020-01299-w 33160335 PMC7648315

[12] Zeltzer A Tonnard P Verpaele A. Sharp-needle intradermal fat grafting (SNIF). *Aesthet Surg J.* (2012) 32:554–61. 10.1177/1090820X12445082 22745443

[13] Fakih-Gomez N Manay R Nazari S Martins L Muñoz-Gonzalez C. Regenerative nanofat membrane development process. *Aesthetic Plast Surg.* (2024) 49:3207–23. 10.1007/s00266-024-04562-5 39663222

[14] Chuchvara N Alamgir M John A Rao B. Dermal filler-induced vascular occlusion successfully treated with tadalafil, hyaluronidase, and aspirin. *Dermatol Surg.* (2021) 47:1160–2. 10.1097/DSS.0000000000002894 33867474

[15] Mehta P Kaplan J Zhang-Nunes S. Ischemic complications of dermal fillers. *Plast Aesthetic Res.* (2022) 9:57. 10.20517/2347-9264.2022.19

[16] Mannino M Lupi E Bernardi S Becelli R Giovannetti F. Vascular complications with necrotic lesions following filler injections: literature systematic review. *J Stomatol Oral Maxillofac Surg.* (2023) 125:101499. 10.1016/j.jormas.2023.101499 37178872

[17] Yi L Chien S. Early recognition of vascular complication following hyaluronic acid filler injection to prevent inadvertent tissue necrosis. *J Asia Pac Aesthetic Sci.* (2023) 3.

[18] Ors S. The effect of hyaluronidase on depth of necrosis in hyaluronic acid filling-related skin complications. *Aesthetic Plast Surg.* (2020) 44:1778–85. 10.1007/s00266-020-01759-2 32424534

[19] Jones D Fitzgerald R Cox S Butterwick K Murad M Humphrey S Preventing and treating adverse events of injectable fillers: evidence-based recommendations from the American society for dermatologic surgery multidisciplinary task force. *Dermatol Surg.* (2021) 47:214–26. 10.1097/DSS.0000000000002921 33543879

[20] Hong G Hu H Chang K Park Y Lee K Chan L Adverse effects associated with dermal filler treatments: part II vascular complication. *Diagnostics (Basel).* (2024) 14:1555. 10.3390/diagnostics14141555 39061692 PMC11276034

[21] Fakih-Gomez N Porcar Plana C Verano-Garcia A Muñoz-Gonzalez C Kadouch J. Updated filler emergency kit: next-generation emergency solution. *Aesthetic Plast Surg.* (2024) 48:1174–80. 10.1007/s00266-023-03722-3 37957396

[22] Farmer A Murray G Croasdell B Davies E Convery C Walker L. Facial vascular events and tissue ischemia: a guide to understanding and optimizing wound care. *J Clin Aesthet Dermatol.* (2021) 14:S39–48. PMC8903221 35291261

[23] Hassan A Chappell A Boyd R Joshi C Wan R Carabano M The use of botulinum toxin to prevent anastomotic thrombosis and promote flap survival: a bridge to developing clinical studies. *Ann Plast Surg.* (2021) 87:222–9. 10.1097/SAP.0000000000002666 33470625

[24] Shi S Jin R Huang C Zhou J. Effect of botulinum toxin type A on flap surgery in animal models: a systematic review and meta-analysis. *J Plast Surg Hand Surg.* (2022) 56:198–207. 10.1080/2000656X.2021.1953044 34338133

[25] Źebryk P Puszczewicz M. Botulinum toxin A in the treatment of Raynaud’s phenomenon: a systematic review. *Arch Med Sci.* (2016) 12:864–70. 10.5114/aoms.2015.48152 27478469 PMC4947604

[26] Nazari S Hadadian N Bayat F Pourani M Muñz-Gonzalez C Fakih-Gomez N. The effects of botulinum toxin on vascular diameter: a preliminary report. *J Cosmet Dermatol.* (2025) 24:e70113. 10.1111/jocd.70113 40071587 PMC11898144

[27] Hu L Feng Y Liu W Jin L Nie Z. Botulinum toxin type A suppresses arterial vasoconstriction by regulating calcium sensitization and the endothelium-dependent endothelial nitric oxide synthase/soluble guanylyl cyclase/cyclic guanosine monophosphate pathway: an in vitro study. *Exp Biol Med (Maywood).* (2019) 244:1475–84. 10.1177/1535370219878143 31547684 PMC6900707

[28] Moreno Rozo N Gómez Díaz O Beltran Pachon P Restrepo D Gómez S. Vascular effects of botulinum toxin in human skin. *Plast Reconstr Surg Glob Open.* (2025) 13:e6384. 10.1097/GOX.0000000000006384 39877206 PMC11774267

[29] Goldberg S Akoon A Kirchner H Deegan J. The effects of botulinum Toxin A on pain in ischemic vasospasm. *J Hand Surg Am.* (2021) 46:513.e1–12. 10.1016/j.jhsa.2020.11.005. 33431193

[30] Rauso R Zerbinati N Franco R Chirico F Ronchi A Sesenna E Cross-linked hyaluronic acid filler hydrolysis with hyaluronidase: different settings to reproduce different clinical scenarios. *Dermatol Ther.* (2020) 33:e13269. 10.1111/dth.13269 32061001

[31] DeLorenzi C. Transarterial degradation of hyaluronic acid filler by hyaluronidase. *Dermatol Surg.* (2014) 40:832–41. 10.1097/DSS.0000000000000062 25022707

[32] Frank S Lalonde D. How acidic is the lidocaine we are injecting, and how much bicarbonate should we add? *Can J Plast Surg.* (2012) 20:71–3. 10.1177/229255031202000207 23730153 PMC3383550

[33] Jung H. Hyaluronidase: an overview of its properties, applications, and side effects. *Arch Plast Surg.* (2020) 47:297–300. 10.5999/aps.2020.00752 32718106 PMC7398804

[34] Ugo U Paola M Salvatore F Giovanni M. Use of minimal amounts of hyaluronidase in the ultrasound-guided treatment of acute vascular occlusion by hyaluronic acid: a preliminary report. *Aesthet Surg J Open Forum.* (2024) 6:ojae025. 10.1093/asjof/ojae025 38938923 PMC11210066

[35] Zhang L Feng X Shi H Wu W Wu S. Blindness after facial filler injections: the role of extravascular hyaluronidase on intravascular hyaluronic acid embolism in the rabbit experimental model. *Aesthet Surg J.* (2019) 40:319–26. 10.1093/asj/sjz280 31630161

[36] Nie F Xie H Wang G An Y. Risk comparison of filler embolism between polymethyl methacrylate (PMMA) and hyaluronic acid (HA). *Aesthetic Plast Surg.* (2019) 43:853–60. 10.1007/s00266-019-01320-w 30824948 PMC6522461

[37] Zhuang J Zheng Q Su X Jiang L Hu J. Clinical manifestations and prognosis of embolism caused by filler injection in different facial regions. *Plast Reconstr Surg Glob Open.* (2023) 11:e5225. 10.1097/GOX.0000000000005225 37650096 PMC10465098

[38] Bilgen F Ural A Bekerecioglu M. Platelet-rich fibrin: an effective chronic wound healing accelerator. *J Tissue Viability.* (2021) 30:616–20. 10.1016/j.jtv.2021.04.009 34275723

[39] Zhang T Wang J Le K Guo Y Zhu B. Platelet-rich fibrin accelerates skin wound healing in pressure injuries: a rat model. *J Wound Care.* (2022) 31:800–4. 10.12968/jowc.2022.31.9.800 36113546

[40] Cl K Jeyaraman M Jeyaraman N Ramasubramanian S Khanna M Yadav S. Antimicrobial effects of platelet-rich plasma and platelet-rich fibrin: a scoping review. *Cureus.* (2023) 15:e51360. 10.7759/cureus.51360 38292974 PMC10825076

[41] Choukroun J Diss A Simonpieri A Girard M Schoeffler C Dohan S Platelet-rich fibrin (PRF): a second-generation platelet concentrate. Part IV: clinical effects on tissue healing. *Oral Surg Oral Med Oral Pathol Oral Radiol Endod.* (2006) 101:e56–60. 10.1016/j.tripleo.2005.07.011 16504852

[42] Soares D. Bridging a century-old problem: the pathophysiology and molecular mechanisms of HA filler-induced vascular occlusion (FIVO)-implications for therapeutic interventions. *Molecules.* (2022) 27:5398. 10.3390/molecules27175398 36080164 PMC9458226

[43] Miron R Fujioka-Kobayashi M Bishara M Zhang Y Hernandez M Choukroun J. Platelet-rich fibrin and soft tissue wound healing: a systematic review. *Tissue Eng Part B Rev.* (2017) 23:83–99. 10.1089/ten.TEB.2016.0233 27672729

[44] Gushiken L Beserra F Bastos J Jackson C Pellizzon C. Cutaneous wound healing: an update from physiopathology to current therapies. *Life.* (2021) 11:665. 10.3390/life11070665 34357037 PMC8307436

[45] Yu P Zhai Z Jin X Yang X Qi Z. Clinical application of platelet-rich fibrin in plastic and reconstructive surgery: a systematic review. *Aesthetic Plast Surg.* (2018) 42:511–9. 10.1007/s00266-018-1087-0 29396591

[46] Arita A Tobita M. Adverse events related to platelet-rich plasma therapy and future issues to be resolved. *Regen Ther.* (2024) 26:496–501. 10.1016/j.reth.2024.07.004 39100535 PMC11295534

[47] Ousey K Ovens L. Comparing methods of debridement for removing biofilm in hard-to-heal wounds. *J Wound Care.* (2023) 32:S4–10. 10.12968/jowc.2023.32.Sup3b.S4 36971485

[48] Choi WY Cho HW Lee DW. Complications of injectable soft tissue filler. *Arch Aesthetic Plastic Surg.* (2015) 21:1–6. 10.14730/aaps.2015.21.1.1

[49] Sanchez-Macedo N McLuckie M Grünherz L Lindenblatt N. Protein profiling of mechanically processed lipoaspirates: discovering wound healing and antifibrotic biomarkers in nanofat. *Plast Reconstr Surg.* (2022) 150:341e–54e. 10.1097/PRS.0000000000009345 35666150 PMC10231932

[50] Liang Z Lu X Li D Liang Y Zhu D Wu F Precise intradermal injection of nanofat-derived stromal cells combined with platelet-rich fibrin improves the efficacy of facial skin rejuvenation. *Cell Physiol Biochem.* (2018) 47:316–29. 10.1159/000489809 29768259

[51] Yu Q Cai Y Huang H Wang Z Xu P Wang X Co-transplantation of nanofat enhances neovascularization and fat graft survival in nude mice. *Aesthet Surg J.* (2018) 38:667–75. 10.1093/asj/sjx211 29161346

[52] Menkes S Luca M Soldati G Polla L. Subcutaneous injections of nanofat adipose-derived stem cell grafting in facial rejuvenation. *Plast Reconstr Surg Glob Open.* (2020) 8:e2550. 10.1097/GOX.0000000000002550 32095390 PMC7015601

[53] La Padula S Ponzo M Lombardi M Iazzetta V Errico C Polverino G Nanofat in plastic reconstructive, regenerative, and aesthetic surgery: a review of advancements in face-focused applications. *J Clin Med.* (2023) 12:4351. 10.3390/jcm12134351 37445386 PMC10342690

